# Challenges to infection control in early communication intervention: A scoping review

**DOI:** 10.4102/sajcd.v69i2.911

**Published:** 2022-08-03

**Authors:** Bilqees Achmat, Berna Gerber

**Affiliations:** 1 Division of Speech-Language and Hearing Therapy, Faculty of Medicine and Health Sciences, Stellenbosch University, Tygerberg, South Africa

**Keywords:** early communication intervention, early intervention, personal protective equipment, infection prevention and control measures, infection control, scoping review, PRISMA-ScR

## Abstract

**Background:**

Personal protective equipment (PPE) and infection prevention and control (IPC) measures are crucial to preventing the spread of coronavirus disease 2019 (COVID-19). This study used a scoping review to investigate the challenges that exist when speech–language therapists (SLTs) use IPC measures for providing early communication intervention (ECI).

**Objectives:**

To describe existing, recent literature on PPE and IPC measures used in early intervention through a scoping review (steps 1–5) and to consult local clinicians to investigate how SLTs who provide ECI in South Africa relate to these findings (step 6 of the scoping review).

**Method:**

A scoping review was performed which followed the PRISMA-ScR framework. Because of limited literature on PPE and IPC measures used by SLTs in providing ECI, the inclusion criteria were adjusted to include PPE and IPC measures used by healthcare workers (HCWs) who provide early intervention to the population of infants and toddlers up to 3 years old. At the time of the review, articles were not older than 10 years and were published between 2011 and 2020. The scoping review included a consultation with South African SLTs who provide ECI, including during the COVID-19 pandemic. A pilot study was conducted prior to the consultations. Seventeen clinicians were included in total. Data from both the pilot study and main consultation were transcribed and analysed in the results using thematic analysis.

**Results:**

Fourteen articles were included in the study. The scoping review of existing literature identified challenges to implementing IPC measures, namely the care and behaviour of young children, infrastructure and system challenges, poor compliance and lack of training and a lack of standard IPC protocols. Clinicians in the consultation phase confirmed these challenges and reported that IPC measures did not consider ECI populations nor the settings in which services were provided. Suggestions from the literature for improved infection control included hand hygiene, improved supplies and infrastructure and education and training. Clinicians in the consultation added practical suggestions for implementing IPC measures within ECI, which included an increase in parent-led intervention as well as cleaning and disinfection strategies.

**Conclusion:**

This study identified challenges and recommendations of SLTs who use PPE and IPC measures whilst providing ECI. Understanding these challenges can benefit ECI services and future research efforts focused on improving ECI services whilst maintaining IPC standards.

## Introduction

The coronavirus disease 2019 (COVID-19) pandemic has disrupted healthcare services around the world (Manto et al., 2020). Speech–language therapists (SLTs) who provide early communication intervention (ECI) have faced exceptional challenges during the pandemic because of the nature of ECI, which includes face-to-face exchanges and close proximity to patients (Owens, 2016). As a result, SLTs providing ECI and their clients are at risk of exposure to infectious agents. One clear strategy to reduce the spread of COVID-19 is the use of personal protective equipment (PPE) and infection prevention and control (IPC) measures (Sarma et al., 2020). Personal protective equipment may, however, disrupt ECI services, as face masks obstruct visual access to the mouth and can reduce speech perception (Atcherson et al., 2017; Hashikawa et al., 2020; Khan et al., 2019). In addition, implementing IPC measures may be particularly challenging in ECI as young children are unable to perform adequate self-hygiene without supervision (Hashikawa et al., 2020). Thus, a balance is needed between the optimal delivery of ECI services and the maintenance of IPC standards.

For SLTs to devise the best strategies for implementing IPC measures as well as providing effective ECI services, the existing PPE and IPC measures used within ECI need to be identified and described. A review of existing literature revealed no recommendations for the best practice of IPC measures by SLTs within ECI. There is also limited available literature on IPC measures used by other healthcare workers (HCWs) within early intervention for children from birth to 3 years. Furthermore, most of the relevant studies were conducted in high-income countries (HICs) (Buser et al., 2013; Gupta & Pursley, 2011; Verma et al., 2020; Yin, Schweizer, Herwaldt, Pottinger, & Perencevich, 2013). This challenges the transferability of research findings in the relevant literature to settings in low- to middle-income (LMICs) countries. For example, in LMICs such as South Africa, the implementation of IPC measures may be restricted by the lack of available and affordable PPE as well as insufficient healthcare infrastructure (Blignaut, Nemutandani, & Samaranayake, 2020; Sokunbi et al., 2020). Infection prevention is essential for providing high-quality and safe healthcare services to vulnerable populations such as neonates (Dramowski et al., 2020).

The aim of this study was, firstly, to identify the PPE and IPC measures used in early intervention as reported in existing and recent literature and, secondly, to investigate how local clinicians (SLTs who provide ECI in South Africa) relate to the scoping review findings. This was achieved through two research objectives: (1) to describe the recent research literature on PPE and IPC measures used in early intervention through steps 1–5 of a scoping review and (2) to reflect on these literature review findings through consultations with South African SLTs involved in ECI, as step 6 of the scoping review.

### Early communication intervention

‘Early intervention’ refers to services provided by HCWs to the population of birth to 3-year-old infants, toddlers and their caregivers (South African Speech-Language-Hearing Association, 2017). ‘Early communication intervention’ is distinguished from early intervention in that it focuses on communication and language skills and is provided by SLTs (South African Speech-Language-Hearing Association, 2017).

During ECI, SLTs are often in close proximity to young children (e.g. when playing, feeding and cleaning) and are exposed to bodily fluids and secretions (e.g. drooling, mouthing, crying, uncontrolled respiratory secretions and incontinence), which places SLTs at risk of exposure to potential infectious agents (Hashikawa et al., 2020; Siegel, Rhinehart, Jackson, & Chiarello, 2007). As young children are not able to adopt the same infection control practices as adults, it is essential that practitioners observe the proper use and disposal of PPE and IPC measures to protect both vulnerable patients and staff.

### Personal protective equipment and infection prevention and control measures

Personal protective equipment refers to barriers and respirators that are used to reduce contact between infectious agents and airways, skin and clothing. There are different types of PPE, including gloves, gowns, face masks, goggles, face shields and respiratory protection. The PPE is selected according to the most likely mode of transmission and nature of HCW–patient interaction (Siegel et al., 2007).

Infection prevention and control measures are defined as actions that inhibit or reduce the spread of disease within an environment (Khan et al., 2019). Standard precautions should be used for all patients, and additional transmission-based precautions should be used for the management of droplets, close contact and airborne transmissions (Siegel, 2007). Adherence to IPC measures may include implementing teamwork, education and training, screening of patients, hand hygiene, disinfection and isolation of the ill (Khan et al., 2019; Northway, Langley, & Skippen, 2011; Siegel et al., 2007).

### Challenges

The COVID-19 pandemic has compelled HCWs to adopt certain PPE and IPC measures to combat the spread of the disease. This includes wearing face masks, maintaining social distancing, following respiratory etiquette (covering a cough) and handwashing (Chu et al., 2020; Hashikawa et al., 2020). These PPE and IPC measures may present challenges to SLTs providing ECI. For example, face masks cover visual cues and expressions (such as smiles, frowns and smirks) which are crucial when targeting pre-linguistic skills in ECI (Atcherson et al., 2017; Keen et al., 2016). In addition, social distancing may cause speech to be less audible or even completely unheard (Grieco-Calub, 2021).

Furthermore, infants and toddlers can bring about barriers to implementing IPC measures. For example, ECI involves close physical interactions with young children who may not practise proper cough etiquette, and this increases the need for respiratory protection (Northway et al., 2011; Siegel et al., 2007; South African Speech-Language-Hearing Association, 2017). In addition, young children often expel bodily fluids and secretions when held; however, there is little guidance on the frequency of changing gloves and gowns. Lastly, young children frequently mouth their hands and touch surfaces. This requires repeated disinfection of surfaces, which can be challenging in settings with limited time and resources (Hashikawa et al., 2020).

There are strategies that can be implemented to alleviate these challenges. Firstly, coaching parents is an effective method of providing ECI, which can reduce close contact encounters with young children. However, ECI consists of multiple components, of which parent-led intervention is only one (Dathan Rush, 2021). Secondly, virtual communication or telecommunication is recommended as a practical and efficient method of providing care during the social distance restrictions of the COVID-19 pandemic (Sarma et al., 2020). However, virtual communication cannot fully replace in-person encounters, and individuals in LMICs may have technological constraints and/or difficulty accessing networks and the Internet (Manto et al., 2020; Sokunbi et al., 2020).

### Context

In South Africa, the majority of children live in underserved and disadvantaged communities. These children are at increased risk for communication delay because of poverty, disease and psychosocial hazards (Du Toit, Van Der Linde, & Swanepoel, 2021; Samuels et al., 2012). In addition, children in low-resource settings are at increased risk of developing healthcare-associated infections (HAIs) because of host factors (e.g. human immunodeficiency virus [HIV] and acquired immunodeficiency syndrome [AIDS], malnutrition) and health system factors (e.g. poor infrastructure and lack of IPC practices) (Rothe, Schlaich, & Thompson, 2013). Consequently, there is a high demand for ECI services which implement efficient IPC measures.

There is limited research on the implementation of IPC measures by South African SLTs during the COVID-19 pandemic (Adams, Seedat, Coutts, & Kater, 2021). A study by Adams et al. (2021) highlighted the impact of COVID-19 on the services delivered by SLTs. Although the study did not focus on ECI, the findings indicated that paediatric and high-risk patients were most impacted by disrupted outpatient services during the pandemic. Furthermore, Blignaut et al. (2020) described South Africa’s response capacity to the pandemic in comparison to HICs. South Africa was described as having additional challenges such as a lack of resources (e.g. PPE) and chronic infrastructure challenges (e.g. regular electricity and water shortages) (Blignaut et al., 2020). Challenges to implementing infection control existed in South Africa prior to the COVID-19 pandemic. According to Dramowski, Cotton and Whitelaw (2017), previously existing environmental challenges to HAI prevention in neonates and children included overcrowding, high patient-to-staff ratios, lack of IPC provision, lack of isolation facilities, inadequate cleaning and reuse and sharing of equipment. These previous inadequacies in healthcare have most likely worsened because of the COVID-19 pandemic (Sokunbi et al., 2020). The purpose of this study was to describe PPE and IPC measures used in early intervention through a scoping review of existing and recent literature and consultation with South African SLTs who provide ECI.

## Methods

This study followed a scoping review design and comprised six steps in accordance with existing methodological guidance (Arksey & O’Malley, 2005; Levac, Colquhoun, & O’Brien, 2010; Aromataris & Munn, 2020). The scoping review consisted of a systematic literature search (steps 1–5) followed by a consultation with local clinicians (step 6). The consultation took the form of online focus group discussions (FGDs).

The Preferred Reporting Items for Systematic Reviews and Meta-Analyses Extension for Scoping Reviews (PRISMA-ScR) was used as a reporting guideline to ensure methodological transparency in the scoping review (Tricco et al., 2018; see Appendix 1). In addition, the Joanna Briggs Institute (JBI) (Aromataris & Munn, 2020) recommends cross-checking the synthesised findings of the scoping review to assign a level of credibility. Thus, stages 1–5 of the scoping review were cross-checked by a research assistant (RA) with knowledge of and experience in scoping reviews as well as ECI.

### Step 1: Identifying the research question

According to Levac et al. (2005), the purpose of the scoping review should be considered whilst articulating the research question. The purpose of this scoping review was to describe PPE and IPC measures used in early intervention as reported in the current literature and to reflect on the scoping review findings by consulting with stakeholders, namely South African SLTs involved in ECI. Therefore, the research question and sub-question were as follows: (1) what is known from the existing international literature about the available PPE and IPC measures used within early intervention? and (1.1) how do SLTs in South Africa who provide ECI relate to the literature (scoping) review findings with regard to PPE and IPC measure used for ECI?

### Step 2: Identifying relevant studies

The JBI’s (Peters et al., 2020) three-step iterative search strategy was used to guide the identification of relevant studies. Firstly, a search of the topic was conducted on nine online databases. This was followed by an analysis of the text words in the title, abstract and index terms used to describe the retrieved sources. A second search was then conducted across 13 databases using the identified keywords and index terms. The databases included Clinical Key, Cochrane Library, PubMed, Science Direct, Scopus, Web of Science, Google Scholar, University of Stellenbosch Library, Sabinet African Journals, African Journals Online, African Digital Repository Web of Science, African Education Research Database and EBSCOhost Research Databases.

Because of the extensive number of keywords used across studies, 16 different combinations of search strategies were developed. An example of the combination of keywords included [‘personal protective equipment’ OR ‘personal protection equipment’ OR ‘risk control equipment’] AND [‘infection prevention and control measures’ OR ‘infection control’] AND [‘health care professionals’ OR ‘health care providers’ OR ‘health care workers’ OR ‘health care staff’] AND [‘early childhood intervention’ OR ‘early childhood development’ OR ‘early communication intervention’]. Then, a third search was undertaken using the reference lists of identified sources. Professionals and scholars in the field were also contacted to provide any additional relevant literature not identified through the database searches (Peters et al., 2020).

### Inclusion criteria for the selected studies

Any existing literature (published or unpublished) which met the inclusion criteria were accepted, for example, primary research studies, reviews, letters, guidelines, meta-analyses, websites and books. The selected studies were those which:

- were written in English
- were not older than 10 years at the time of the search and published between 2011 and 2020
- focused on early intervention services provided by HCWs
- focused on services delivered to the population of children from birth to 3 years
- focused on PPE and/or IPC measures in delivering the early intervention services
- were available through the University of Stellenbosch library service.

### Step 3: Study selection

The first author independently screened the titles of the search results, and the collected sources were cross-checked by the RA. Then, both the first author and the RA conducted an abstract and full-text review. The inclusion of studies within the data set was discussed upon the completion of each screening process. The suitability of each study was determined by the inclusion criteria. In instances where a consensus could not be reached, a successive screening process was conducted. For example, the abstract review was succeeded by a full-text review, which was then succeeded by a preliminary data extraction exercise. Studies were included if the information provided in the data extraction process was sufficient to answer the research question.

### Step 4: Charting the data

The data charting process was iterative and included an initial trial to ensure that all relevant results were extracted through the data charting form (Levac et al., 2010; Peters et al., 2020). The final data chart included space to record the authors, year of publication, source, country where the research was conducted, research design, research setting, PPE and/or IPC measure(s) described, function of or rationale for the PPE and IPC measures, person or team involved, age range of children, duration of data collection, aims, results, limitations and recommendations (see Appendix 2). The RA cross-checked the data extracted from a random selection of 28.57% of the selected studies. This was above Naudé and Bornman’s (2018) recommended cross-checking of 20% of the data to increase reliability. A trial analysis was conducted to reach a consensus on any discrepancies.

### Step 5: Collating, summarising and reporting the results

Numerical analysis was used to describe the characteristics of the studies selected in step 4 (Arksey & O’Malley, 2005; see Appendix 3). Thematic analysis was used for qualitative data to identify, analyse and provide a detailed report on patterns (themes) of meaning (Braun & Clarke, 2006). Braun and Clarke’s (2006) six phases of thematic analysis were utilised and included (1) familiarisation, (2) generating codes, (3) searching for, (4) reviewing, (5) defining themes and (6) producing the report. The RA reviewed the completed thematic analysis of step 5 to ensure that the themes and codes were aligned with the research question and objectives. No changes were required in this regard.

### Step 6: Consultation with local early communication interventionists

A consultation exercise was conducted to share the preliminary scoping review findings (of steps 1–5) with local stakeholders to validate the findings and to inform future research (Levac et al., 2010; Pollock et al., 2021). The target population comprised registered SLTs who provided ECI in South Africa during the COVID-19 pandemic. Participants were selected using the nonprobability sampling technique of critical case sampling. This involved the researcher purposefully selecting individuals based on specific characteristics or inclusion criteria (Onwuegbuzie & Collins, 2007). To be included in the consultation, participants had to:

- be South African SLTs (including community service SLTs) registered with the HPCSA
- provide ECI during the COVID-19 pandemic for a minimum of 3 months (as the pandemic necessitated the use of PPE and IPC measures when providing ECI)
- must have sufficient access to the Internet (as the consultation was conducted online).

Participants were recruited through an online advert to take part in the research. The advert was posted on the social medial platforms Facebook and WhatsApp and was also distributed via e-mail. Speech–language therapists who were interested in participating in the study were required to complete an eligibility survey based on the inclusion criteria.

Participants were required to complete an informed consent form before they were included in the study. Twelve participants took part in one of two online FGDs. The FGDs included a 20-minute presentation of the preliminary findings of steps 1–5 of the scoping review as well as a 60-minute semistructured group interview, both conducted by the first author (B.A.). The second author (B.G.) attended both online FGDs to provide technical or any other support required. The interview questions were directly related to the scoping review findings (see Appendix 3). This enabled the researcher to describe the extent to which South African SLTs related to the research findings in terms of their own experiences, challenges and perceptions.

Prior to the online FGDs, a face-to-face pilot study was undertaken by the first author with five SLTs to determine the suitability of the presentation and interview questions, as well as to become more familiar with the process of facilitating FGDs. The pilot study also provided information on possible biases within the FGD design (Naudé & Bornman, 2018). This was done by using an anonymous feedback questionnaire which enabled the pilot study participants to comment on the presentation and FGD questions and provide suggestions for improvement. The feedback resulted in minimal changes to the presentation (e.g. repeating abbreviations and reducing the pace of the presentation) and no changes to the FGD interview questions.

Data from the pilot study were included in the results of the scoping review, along with the main FGD findings. Therefore, a total of 17 clinicians took part in the consultation phase. Clinicians were all registered SLTs who provided ECI within different provinces of South Africa, in both the private and public sectors. All clinicians had at least 3 months’ ECI work experience during the COVID-19 pandemic, which necessitated the use of PPE and IPC measures when providing ECI.

The online and pilot FGDs were transcribed and analysed using thematic analysis. A theoretical (deductive) approach to thematic analysis was followed (Braun & Clarke, 2006) as the transcripts were analysed based on the findings of steps 1–5 of the scoping review. ATLAS.ti version 9 software was used to aid the data coding process and create visual representations of the data. A summary of the results was sent to participants as a form of member checking to increase the credibility of the research findings (Varpio, Ajjawi, Monrouxe, O’Brien, & Rees, 2017). Out of the 17 participants included in the study, 13 responded to the member checking. All clinicians who responded agreed with the research findings and no changes were suggested by the clinicians.

### Ethical consideration

Ethical approval to conduct the study was obtained from the Health Research Ethics Committee (HREC) of Stellenbosch University (ethical clearance number S21/03/047).

## Results

### Selection of sources

**FIGURE 1:**
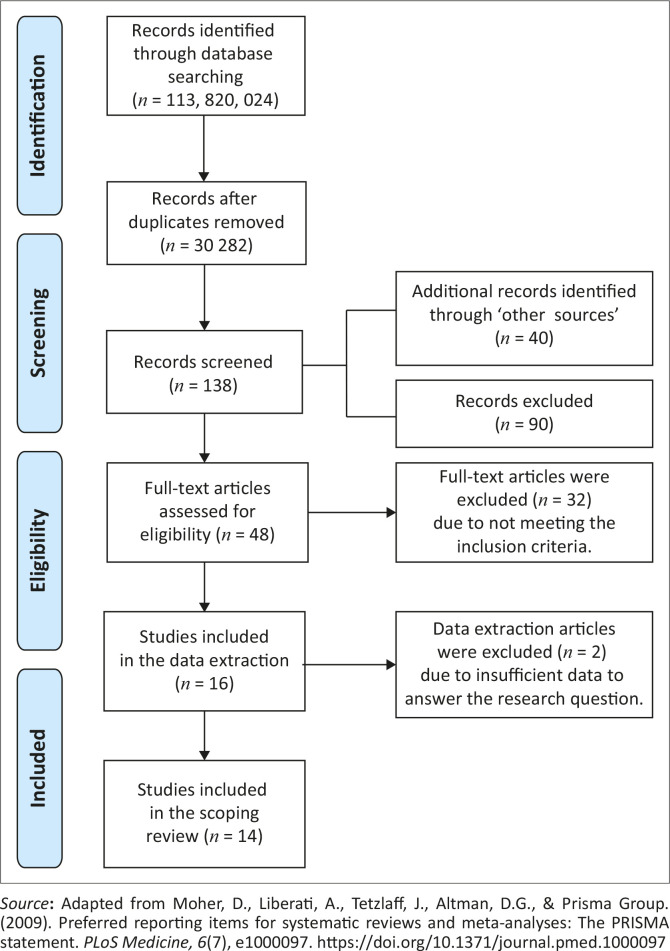
PRISMA-ScR flow diagram of results.

The literature search and selection of sources are depicted in a PRISMA-ScR flow diagram (Figure 1). There were 113 820 024 sources identified in the initial search across 13 online databases. A total of 30 282 studies were screened after duplicates were removed. ‘Other sources’ included the search of reference lists, which resulted in the identification of 31 studies, as well as contact with relevant professionals, which provided an additional nine studies. Within the data extraction, more than half (nine) of the studies were obtained through ‘other sources’. This demonstrates the importance of expanding the literature search beyond electronic databases.

### Characteristics of the included studies

Appendix 4 represents the characteristics of the 14 studies included in the scoping review. Studies were published over a 10-year period between 2011 and 2020. There was no relevant grey literature identified for inclusion in the study. According to the World Bank Classification (ChartsBin Statistics Collector Team, 2016), the majority of studies (*n* = 9) were conducted in HICs. Furthermore, the only study to include a nonmedical setting was that of Ibfelt, Engelund, Schultz and Andersen (2015).

There were no studies identified which discussed PPE and IPC measures used by SLTs in providing ECI. The included studies all discussed PPE and IPC measures used by HCWs who provided early intervention to the population of birth to 3-year-old infants and toddlers. More than half (*n* = 8) of the studies included a team of HCWs, which suggests that the task and responsibility of carrying out IPC measures was well distributed amongst different individuals within the relevant healthcare settings. However, caregivers, who are integral to early intervention services (Owens, 2016), were only included in one study (Buser, Fisher, Shea, & Coffin, 2013). The selected studies focused significantly more on IPC measures than on PPE. This has a bearing on the nature of challenges reported in the selected studies in relation to early intervention.

### Thematic results

In this scoping review, the main results of the literature search (steps 1–5) included challenges and suggestions to implementing IPC measures within early intervention by different HCWs. During the consultation exercise, practitioners related all of the literature search findings to their professional experience and shared additional challenges and suggestions relating to infection control used within ECI, with relevance to the South African context. The combined results of the literature review and consultation phase revealed three core themes, namely challenges to establishing IPC measures within early intervention, challenges to conducting ECI when utilising IPC measures and overcoming the challenges. The results of the literature search and consultation are concurrently presented and discussed below. The identity of stakeholders in the consultation phase has been anonymised by the code P (pilot study), F1 (FGD 1) or F2 (FGD 2) followed by the p (participant) number; for example Pp1 refers to Participant 1 in the pilot study.

## Discussion

### Theme 1: Challenges to establishing infection prevention and control measures within early intervention

The literature search highlighted several challenges to establishing IPC measures within early intervention. During the consultation, practitioners reflected on these findings in relation to the ECI services they provide in South Africa. The challenges included five major barriers to implementing IPC measures.

#### The nature of care and behaviour of young children

**FIGURE 2:**
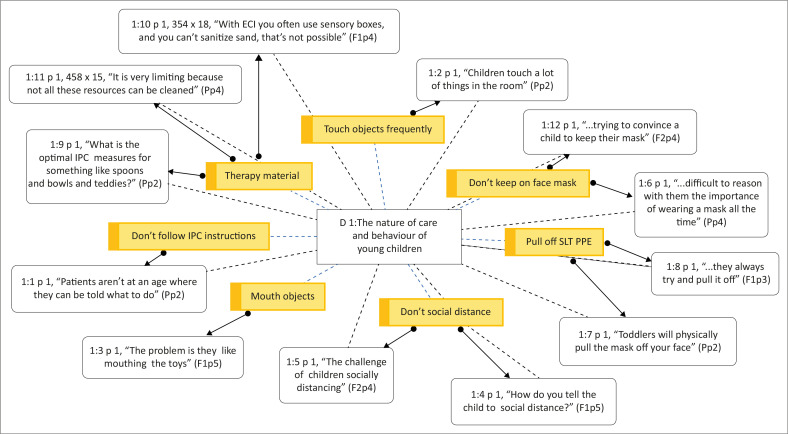
Organic network showing the nature of care and behaviour of young children in response to infection prevention and control measures within early communication intervention as reported in the focus group discussions, generated by ATLAS.ti version 9.

Young children receiving ECI are not developmentally capable of understanding and practising infection control without caregiver supervision (Hashikawa et al., 2020). Only one of the selected studies in the literature review included caregivers in IPC practices (Buser et al., 2013). Moreover, none of the studies considered the nature of care and behaviour of young children. On the other hand, practitioners in the consultation frequently described barriers to implementing IPC measures because of the nature and behaviour of young children. Participants stated that young children frequently touch surfaces, mouth objects, pull off their face masks, pull off the practitioner’s face mask and have difficulty with social distancing (see Figure 2). In addition, toys are often used in intervention (Koutlakis-Barron & Hayden, 2016). The practitioners reported that certain toys (e.g. ‘teddies’ [Pp2], ‘playdough’, ‘sand’ [F1p4]) and feeding kits (e.g. ‘spoons and bowls’ [Pp2]) were often difficult to disinfect and not easily replaced.

#### Infrastructure and system challenges

In the literature search, infrastructure and system challenges were the most frequently occurring barriers to IPC measures within early intervention. These challenges included overcrowded wards, understaffing, lack of isolation facilities, inadequate ventilation, a lack of time to apply infection control standards, a lack of screening procedures and resource limitations such as poor access to PPE (Dramowski, Whitelaw, & Cotton, 2016; Gupta & Pursley, 2011; Olivier et al., 2018; Reid et al., 2011; Salem & Youssef, 2017; Triantafillou, Kopsidas, Kyriakousi, Zaoutis, & Szymczak, 2020; Verma et al., 2020).

During the consultation, practitioners reported that healthcare facilities had *poor* rather than lacking screening procedures. Practitioners reported that screening procedures were often ‘not strictly enforced’ (F2p2) and unreliable because of ‘false information’ (Pp3) provided by caregivers.

‘I have experienced with some patients [*that*] they lie during the screening procedure just to get into the healthcare facility.’ (F2p2)

Practitioners based in nonmedical settings (such as ‘private practices … schools …homes’ [F2p6]) related to the lack of funding for PPE and added that PPE was not covered by patients’ medical aid schemes. Moreover, IPC measures consumed session time which could not be billed. Therefore, both the cost and time of implementing IPC measures were financial burdens. In the words of F2p6, ‘how do you balance the time sanitising and therapy and then billing for that?’ Practitioners considered these challenges to be more prevalent in LMICs, such as South Africa, in comparison to HICs.

Most studies included in the scoping review were from HICs (*n* = 9), which almost certainly have a greater availability of resources and technology compared to LMICs (Scherzer, Chhagan, Kauchali, & Susser, 2012). Participants commented that more research is required on infection control within ECI in LMICs, as IPC measures designed in HICs may not be easily generalised to LMICs.

#### Differences in infection prevention and control measures between medical and nonmedical settings

Only one of the selected studies in the scoping review was based in a nonmedical setting (Ibfelt et al., 2015), which suggests a shortage of research in this regard. During the consultation practitioners related to the need for greater awareness of IPC practices beyond medical settings. As SLTs often work in a variety of different locations (Pillay & Pillay, 2021), they were able to list key differences in infection control between these two settings. In comparison to medical settings, nonmedical settings were stated to have less ‘access to information’ (F2p6) regarding infection control, less ‘variety of PPE’ (Pp1) and a lack of standard IPC ‘protocols’ (F2p6).

In addition, practitioners regarded the risk of cross-contamination to be greater in nonmedical settings because of working in more than one location and the patients’ exposure to multiple contacts (e.g. peers, teachers and family). In contrast, medical settings might have isolation cubicles or restricted contact between patients and visitors, which limits the risk of cross-contamination beyond the one setting:

‘[*T*]hey’re in contact with so many other kids and teachers and parents. Whereas your inpatient client, sometimes they’re in their own room where they don’t have a lot of contact with other people.’ (F1p3)

#### Poor infection prevention and control compliance amongst healthcare workers, patients and caregivers

Poor IPC practices by HCWs were also frequently reported in the selected studies. Siegel et al. (2007) stated that adherence to IPC measures amongst HCWs can range between 43% and 89%. The comments during the consultation expanded on poor IPC compliance to include patients and caregivers:

‘The staff and everyone can have the most amazing PPE and hygiene and cleaning. But if the parent doesn’t comply, then they are even putting us at risk for further infections.’ (Pp1)

Education and training have been associated with improvements to IPC compliance (Siegel et al., 2007). However, a lack of IPC training for staff members and caregivers was also identified as a barrier and is discussed below.

#### Lack of infection prevention and control training for staff members and caregivers

The literature review identified a lack of training for HCWs as a barrier to compliance with IPC measures. The study by Triantafillou et al. (2020) reported a lack of continued education and training on infection control, starting with undergraduate education at university. Practitioners taking part in the consultation agreed with this finding and further motivated for the IPC training of caregivers:

‘With the caregivers … we all have that responsibility in teaching, training, empowering. To tell them why it’s important to understand, and then I think they’ll comply much easier.’ (Pp2)

The importance of training caregivers on infection control within ECI is twofold. Speech-language therapists should include caregivers in ECI services, because young children will imitate the behaviour of their parents (Owens, 2016). At the same time, the inclusion of caregivers in IPC training is important because young children do not practise adequate infection control without adult supervision (Koutlakis-Barron & Hayden, 2016). Buser et al. (2013) investigated the inclusion of parents in the infection control programme of a hospital facility. The results indicated that parents were willing to collaborate with HCWs and that empowering parents to participate in infection control practices can improve child health.

### Theme 2: Challenges to conducting early communication intervention when utilising infection prevention and control measures

**FIGURE 3:**
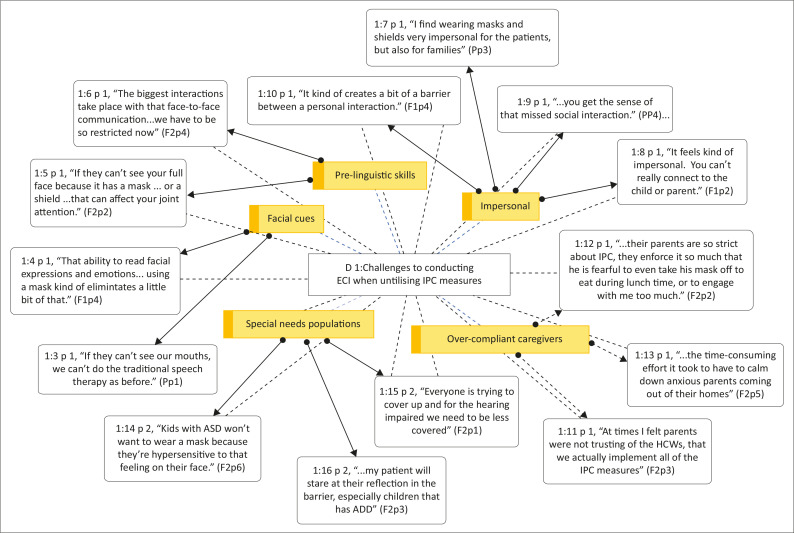
Organic network showing the challenges to conducting early communication intervention when utilising infection prevention and control measures as expressed during the focus group discussions, generated by ATLAS.ti version 9.

Participants in the consultation described five central challenges to conducting ECI when utilising IPC measures (see Figure 3). These challenges were additional to those found in the literature search and demonstrate the value of conducting a consultation with local stakeholders to determine the applicability of research findings for SLTs in South Africa.

#### Difficulty targeting prelinguistic skills

Firstly, practitioners described difficulty in targeting prelinguistic skills within ECI as face masks and/or face shields cover most of the face. Prelinguistic skills can be seen as the building blocks of communication development and greatly rely on face-to-face exchanges to gain and maintain a child’s interest (Keen et al., 2016; Owens, 2016). Speech–language therapists must work harder and more creatively to motivate infants to participate in episodes of joint attention, whilst still maintaining IPC standards.

#### Difficulty providing facial cues

Secondly, practitioners had difficulty providing visual cues with their mouths when wearing a face mask. Speech–language therapists can use visual cues to increase speech perception or model the phonetic placement of speech sounds for patients to imitate (Atcherson et al., 2017; Owens, 2016). Face masks hinder SLTs’ ability to provide visual cues which can improve speech perception and production (Atcherson et al., 2017). In addition, face masks filter the acoustic speech signal, which can further reduce speech recognition. Certain types of masks may reduce the signal more than others (Grieco-Calub, 2021). It has been recommended that face shields or face masks with transparent windows should be used to provide visual cues and facilitate speech recognition (Thibodeau, Thibodeau-Nielsen, Tran, & De Souza Jacob, 2021). However, clinicians in the consultation reported that face masks with windows and even face shields tend to ‘mist up’ (F1p6), which diminishes their purpose in providing visual access to the mouth. In addition, the material used to manufacture shields and masks with windows may filter acoustic properties worse than surgical or cloth masks (Corey, Jones, & Singer, 2020). Thus, the benefits of using transparent equipment are limited to the extent that they diminish the speech signal.

#### Personal protective equipment is impersonal

Thirdly, practitioners felt that that wearing PPE felt impersonal and created a barrier to developing a relationship or bond with patients and caregivers. According to Van Veenendaal et al. (2021), parents in the Neonatal Intensive Care Unit (NICU) during the COVID-19 pandemic felt that face masks affected bonding with their infants and depersonalised interactions with staff members.

#### Over-compliant caregivers

Fourthly, findings in the literature search indicated a lack of basic infection control education for caregivers, which increases the risk of disease transmission (Buser et al., 2013; Dramowski et al. 2016). However, during the consultation it was reported that since the COVID-19 pandemic, most caregivers are well-informed on IPC measures. In contrast to the existing literature findings, some caregivers were reported to be over-compliant or ‘over-protective’ (F2p3) with regard to infection control. This could be disruptive to the therapy session as IPC practices took time to implement and explain, which resulted in the loss of engagement with patients:

‘At times I felt parents were not trusting of the health care workers, that we actually implement all of the IPC measures … there was a situation where I took about 20 min of my 30 min time slot to explain to the parents that we are hand washing, we are sanitising, we do have our PPE.’ (F2p3)

Moreover, practitioners reported on a few cases where caregivers were reluctant to attend therapy sessions during the COVID-19 pandemic. An effective strategy in these predicaments was the use of *visible* IPC measures such as screening procedures. Visible IPC practices appeared to signify that there were sufficient IPC measures in place for patients to attend ECI services.

‘The hospital had very strict screening protocols at [*the*] entrance, and some parents stated that this put them at ease and [*they*] trusted the IPC measures that were in place. The less-reluctant parents were then happier to come for outpatient therapy, as they felt safe within the hospital.’ (F2p1)

The use of teletherapy is suggested as a strategy to provide healthcare during social distance restrictions (Sarma et al., 2020). None of the studies included in the scoping review mentioned this strategy, and only one participant alluded to the difficulty of interacting with young children over a screen. Teletherapy was, however, mentioned as an effective strategy to inform caregivers of IPC practices in ECI.

‘We had an online meeting … just putting out the disclaimer to say, “this is our practice policies in terms of IPC measures” so that they are little bit more at ease.’ (F2p3)

#### Special needs populations are not considered

Lastly, the most frequently reported challenge reported by practitioners was the lack of consideration for populations with special needs when implementing IPC measures within ECI. Practitioners reported that these populations present with unique characteristics and that the implementation of IPC measures in such cases is often problematic.

‘IPC procedures don’t always take into account the special populations we work with in the ECI field, like our ASD kiddies and kiddies with intellectual impairments that might not understand the IPC procedures as well.’ (F2p6)

### Theme 3: Overcoming the challenges

The importance of hand hygiene was emphasised by all 14 studies in the scoping review. The study by Lorenzini, Costa and Silva (2013) declared hand hygiene to be the most effective and least expensive method for preventing and controlling HAIs. Then, two frequently reported recommendations were improved ‘supplies and infrastructure’ and ‘education and training’ for HCWs and caregivers. Salem and Youssef (2017) reported that without basic supplies and infrastructure, it is not possible for staff members to meet IPC standards or provide an acceptable level of care.

During the consultation, practitioners agreed with these recommendations and provided three additional suggestions to improve IPC practices within ECI. Firstly, practitioners discussed the difficulty of disinfecting intervention material. Cleaning strategies were mentioned such as using ‘different boxes’ (F2*P*3) to indicate the sanitised and unsanitised toys, quarantining therapy material which could not be sanitised such as the ‘sand’ (F1p4) in sensory boxes and placing pictures in ‘plastic sleeves’ (F1p6) so that they could be cleaned more easily. An alternative strategy was to avoid disinfection altogether by asking caregivers to bring therapy material from ‘home’ (F1p1) or to make toys using ‘recyclable materials’ (F1p1) which could easily be disposed of or given to patients after a single use.

Secondly, practitioners reiterated the difficulty of young children not understanding or following IPC instructions. One practitioner suggested having ‘regular hand sanitising times’ (Pp4), especially with children who frequently touch objects and surfaces. Thirdly, the close contact nature of ECI was discussed. Practitioners stated that their approach to ECI had shifted to become more ‘parent-led’ (F2p2) in an attempt to maintain their social distance from the children. This required SLTs to train caregivers on ECI strategies and explain why IPC measures were in place. These modifications were useful in transferring ECI techniques to the patients’ homes and empowering caregivers to take more responsibility for infection control. Parent-led intervention or parent coaching is, of course, the recommended practice to follow in the communication intervention of young children (Dathan Rush, 2021).

The resilience of practitioners in navigating solutions during unprecedented times is commendable, especially considering the lack of standard IPC protocols for ECI. Overall, there was a clear need for ECI-targeted IPC guidelines which take into consideration both the characteristics of the population (e.g. young children with special needs) and the settings in which services are provided (e.g. nonmedical and low-resource settings). The predicament remains of ECI strategies compromising the effectiveness of IPC measures. Understanding the challenges to implementing IPC measures within ECI can be seen as the first step to overcoming them.

## Implications for future research

Further research is needed to determine whether IPC measures and/or ECI strategies can be modified to collectively maintain their effective implementation. The findings of this study echo those of Adams et al. (2021), who described the resilience of SLTs providing effective services in the light of barriers caused by PPE and IPC measures. A related area of research is investigation of the effectiveness of different types of PPE. Existing literature in this field includes the study by Van Der Sande, Teunis and Sabel (2008), who investigated the effectiveness of different face masks in preventing respiratory infections. The benefits of such research to SLTs involve knowing the risk of exposure to infectious agents, as SLTs tend to select PPE which presents the least interference to intervention – for example, wearing a face shield with a transparent cover instead of a face mask which obstructs visual cues. Knowing which PPE is most effective can be useful in low-resource settings, where there is often a need to prioritise limited available supplies. A gap in the research literature includes investigations on the effect of certain PPE on early language development of infants and toddlers. Such research findings would be beneficial to all professionals involved in early intervention to ensure that the best outcome is achieved whilst maintaining IPC standards.

## Limitations

The limitations of this scoping review involved the collection of sources of evidence, which were restricted to studies written in English and studies that did not require paid access. Potentially relevant studies may have been missed because of these restrictions. Moreover, the consultation was with a relatively small number of stakeholders, which reduces the generalisability of the findings. However, the purpose of conducting the consultation was fulfilled in disseminating and validating research findings, as well as identifying research gaps (Levac et al., 2010; Pollock et al., 2021).

## Conclusion

This scoping review utilised a literature search of recent international literature as well as consultation with local clinicians to describe the implementation of PPE and IPC measures within general early intervention and ECI. The findings of the literature review included several challenges faced by HCWs in implementing IPC measures within early intervention. The consultation revealed that SLTs in South Africa faced similar challenges in ECI as those reported in the literature review findings. The SLTs described additional challenges to conducting ECI whilst utilising IPC measures. These challenges included difficulty targeting prelinguistic skills, difficulty providing facial cues, the impersonal impression of PPE, over-compliant caregivers and the lack of consideration for populations with special needs. Limited literature is available on how to overcome the challenges that are specific to ECI. Future research is needed to guide SLTs on the use of PPE and IPC measures within ECI, whilst maintaining the effectiveness of each practice.

## Appendix 1

**TABLE 1-A1:** Preferred Reporting Items for Systematic Reviews and Meta-analyses Extension for Scoping Reviews (PRISMA-ScR) checklist.

Section	Item	PRISMA-ScR checklist item	Reported (Yes/No)
**Title**			
Title	1	Identify the report as a scoping review.	Yes
**Abstract**			
Structured summary	2	Provide a structured summary that includes (as applicable): background, objectives, eligibility criteria, sources of evidence, charting methods, results and conclusions that relate to the review questions and objectives.	Yes
**Introduction**			
Rationale	3	Describe the rationale for the review in the context of what is already known. Explain why the review questions or objectives lend themselves to a scoping review approach.	Yes
Objectives	4	Provide an explicit statement of the questions and objectives being addressed with reference to their key elements (e.g. population or participants, concepts and context) or other relevant key elements used to conceptualise the review questions and/or objectives.	Yes
**Methods**			
Protocol and registration	5	Indicate whether a review protocol exists; state if and where it can be accessed (e.g. a web address), and if available, provide registration information, including the registration number.	N/A
Eligibility criteria	6	Specify characteristics of the sources of evidence used as eligibility criteria (e.g. years considered, language and publication status), and provide a rationale.	Yes
Information sources†	7	Describe all information sources in the search (e.g. databases with dates of coverage and contact with authors to identify additional sources), as well as the date the most recent search was executed.	Yes
Search	8	Present the full electronic search strategy for at least one database, including any limits used, such that it could be repeated.	Yes
Selection of sources of evidence‡	9	State the process for selecting sources of evidence (i.e. screening and eligibility) included in the scoping review.	Yes
Data charting process§	10	Describe the methods of charting data from the included sources of evidence (e.g. calibrated forms or forms that have been tested by the team before their use, whether data charting was done independently or in duplicate) and any processes for obtaining and confirming data from investigators.	Yes
Data items	11	List and define all variables for which data were sought and any assumptions and simplifications made.	Yes
Critical appraisal of individual sources of evidence¶	12	If done, provide a rationale for conducting a critical appraisal of included sources of evidence; describe the methods used and how this information was used in any data synthesis (if appropriate).	N/A
Synthesis of results	13	Describe the methods of handling and summarising the data that were charted.	Yes
**Results**			
Selection of sources of evidence	14	Give numbers of sources of evidence screened, assessed for eligibility and included in the review, with reasons for exclusions at each stage, ideally using a flow diagram.	Yes
Characteristics of sources of evidence	15	For each source of evidence, present characteristics for which data were charted and provide the citations.	Yes
Critical appraisal within sources of evidence	16	If done, present data on critical appraisal of included sources of evidence (see item 12).	N/A
Results of individual sources of evidence	17	For each included source of evidence, present the relevant data that were charted that relate to the review questions and objectives.	Yes
Synthesis of results	18	Summarise and/or present the charting results as they relate to the review questions and objectives.	Yes
**Discussion**			
Summary of evidence	19	Summarise the main results (including an overview of concepts, themes and types of evidence available), link to the review questions and objectives and consider the relevance to key groups.	Yes
Limitations	20	Discuss the limitations of the scoping review process.	Yes
Conclusions	21	Provide a general interpretation of the results with respect to the review questions and objectives, as well as potential implications and/or next steps.	Yes
**Funding**			
Funding	22	Describe sources of funding for the included sources of evidence, as well as sources of funding for the scoping review. Describe the role of the funders of the scoping review.	Yes

*Source:* Tricco, A.C., Lillie, E., Zarin, W., O’Brien, K.K., Colquhoun, H., Levac, D., … Straus, S.E. (2018). PRISMA extension for scoping reviews (PRISMA-ScR): Checklist and explanation. *Annals of Internal Medicine, 169*(7), 467–473. https://doi.org/10.7326/M18-0850

JBI, Joanna Briggs Institute; PRISMA-ScR, Preferred Reporting Items for Systematic reviews and Meta-Analyses Extension for Scoping Reviews.

† , Where sources of evidence (see second footnote) are compiled from, such as bibliographic databases, social media platforms and websites.

‡ , A more inclusive or heterogeneous term used to account for the different types of evidence or data sources (e.g. quantitative and/or qualitative research, expert opinion and policy documents) that may be eligible in a scoping review as opposed to only studies. This is not to be confused with information sources (see first footnote).

§ , The frameworks by Arksey and O’Malley (6) and Levac and colleagues (7) and the JBI guidance (4, 5) refer to the process of data extraction in a scoping review as data charting.

¶ , The process of systematically examining research evidence to assess its validity, results and relevance before using it to inform a decision. This term is used for items 12 and 19 instead of ‘risk of bias’ (which is more applicable to systematic reviews of interventions) to include and acknowledge the various sources of evidence that may be used in a scoping review (e.g. quantitative and/or qualitative research, expert opinion and policy document).

## Appendix 2

**TABLE 1-A2:** Data charting table.

Scoping review details	Data
Authors	
Year of publication	
Source of the selected study	
Country where the research was conducted	
Research design	
Research setting	
PPE and/or IPC measure(s) described	
Function of or rational for the PPE and IPC measures	
Person or team involved	
Age range of children	
Duration of data collection	
Aims	
Results	
Limitations	
Recommendations	

IPC, infection prevention and control; PPE, Personal protective equipment.

## Appendix 3

**TABLE 1-A3:** Consultation phase (step 6): Interview guide.

No.	Description
1.	Within the selected studies, there were two significant spikes in relevant infection control research in early intervention published in 2013 and 2020. The increase and decrease in relevant publications might be linked to the presence or absence of major infectious disease outbreaks.
	Do you agree with this connection, or do you think there is another reason for the increase in research and resulting publications? Please motivate your answer.
2.	Can you describe any challenges to using PPE and maintaining IPC practices which are specific to early communication intervention? Please also mention if you do not experience any challenges.
3.	What would you say the primary function of PPE and IPC measures is if you think about prevention of transmission versus simply protecting oneself?
3.1	Can you share any experiences of how the perceived primary function (protection vs. prevention) affected the use and disposal of PPE by individuals (e.g. staff, patients or caregivers)?
4.	Only one study investigated a childcare facility (e.g. nursery). All other studies were carried out in medical settings. In your work experience, can you describe any similarities or differences between the infection control measures used in medical settings compared to nonmedical settings which provide ECI?
5.	The research findings support a collaborative team approach to infection control. However, only one of the 14 selected studies included caregivers.
5.1.	What are your views on the use of a ‘team approach’ to infection control?
5.2.	What is your experience of including caregivers in IPC strategies within ECI?
6.	To what extent do you relate to the barriers to IPC measures described in the research findings?
6.1.	Do you experience or are you aware of any additional barriers to IPC measures within early communication intervention?
7.	To what extent do you agree or disagree with the recommendations for IPC measures in early intervention service provision, as reported in the scoping review of international literature?
7.1.	Can you suggest any additional recommendations for infection control used in early communication intervention?
8.	What recommendations do you have for future research regarding PPE and IPC measures in ECI provision in countries like South Africa?
9.	Was there something else you would have wanted to know in relation to the research findings?

IPC, infection prevention and control; PPE, Personal protective equipment; ECI, early communication intervention.

## Appendix 4

**TABLE 1-A4:** Characteristics of included studies.

Article #	Author and date	Source	Country and income level	Setting	PPE and/or IPC measures	Person/team involved
1	Triantafillou et al. (2020)	EBSCOhost	Greece (high income)	Medical	IPC measures	Medical staff & nurses
2	Salem and Youssef (2017)	EBSCOhost	Egypt (low income)	Medical	IPC measures	Medical staff & nurses
3	Wong, Xu, Bone, and Srigley (2020)	EBSCOhost	Canada (high income)	Medical	IPC measures	HCWs & hospital teacher
4	Dramowski et al. (2016)	University of Stellenbosch Library	South Africa (middle income)	Medical	IPC measures	Medical staff
5	Reid et al. (2011)	Professionals	Canada (high income)	Medical	PPE & IPC measures	Medical staff
6	Yin et al. (2013)	Professionals	United States of America (high income)	Medical	PPE (gloving)	HCWs
7	Gupta and Pursley (2011)	References	United States of America (High income)	Medical	IPC measures	Medical staff
8	Ibfelt et al. (2015)	References	Denmark (high income)	Nonmedical (nursery)	IPC measures	Nursery carers
9	Gras-Valentí et al. (2020)	References	Spain (high income)	Medical	IPC measures	HCWs
10	Olivier et al. (2018)	References	South Africa (middle income)	Medical	IPC measures	HCWs
11	Lorenzini et al. (2013)	References	Brazil (middle income)	Medical	IPC measures	Nurses
12	Buser et al. (2013)	EBSCOhost	United States of America (high income)	Medical	IPC measures	HCWs & parents
13	Freitas, Alves, and Gaíva (2020)	Google Scholar	Brazil (middle income)	Medical	PPE & IPC measures	HCWs
14	Verma et al. (2020)		United States of America (high income)	Medical	PPE & IPC measures	Medical staff & nurses

IPC, infection prevention and control; HCWs, health care workers; PPE, Personal protective equipment.
